# First-hour protocol clarifies resource management and nursing staff education in the ICU

**DOI:** 10.1186/cc12441

**Published:** 2013-03-19

**Authors:** M Arbelius-Iltanen, R Siren, J Heinilä, P Korhonen, T Sutinen, V Jalkanen, T Ahonen, S Karlsson

**Affiliations:** 1 Tampere University Hospital, Tampere, Finland

## Introduction

The first-hour protocol determines the patient-specific resources for the start of an ICU stay [1]. Staff resources are decided through triage. Task charts direct the start of intensive care. Our primary goal is to improve patient care.

## Methods

A triage method (red, yellow, green) is used to manage ICU resources according to the severity of illness. For example, one doctor and one nurse would admit a stable (green) patient coming from the operating room for postoperative ICU care. A patient in septic shock with multiple organ disorder (red), on the other hand, would be admitted by a team of two doctors and three nurses. Each staff member has a task chart in a checking-list format. Also, an admission chart is used to improve data collection. The use of the protocol started as a pilot study in early 2012. Simulation education for staff members started in August 2012 and has included video recordings and debriefing of each simulated ICU admission. Primary goal-directed therapy goals have been mean arterial pressure (MAP >65 mmHg), SpO_2 _>94%, timing of the laboratory tests, start of antibiotics, and blood glucose level 6 to 8 mmol/l. Quality indicators have been followed from the data provided by The Finnish Intensive Care Consortium. Questionnaires for the staff members have been used to evaluate opinions about the first-hour protocol.

## Results

According to the questionnaire replies, 80% (*n *= 64/80) of our nurses estimate that the first-hour protocol has improved the starting process of our patients' intensive care. Twenty percent (*n *= 16/80) of the nurses considered that the protocol has no effect, and none thought it to be adverse for patient care. Corresponding numbers for our ICU doctors were 87% (beneficial *n *= 13/15), 13% (no effect *n *= 2/15) and 0% (adverse). Furthermore, 82.5% (*n *= 66/80) of the nurses replied that education of new nurse staff members has improved because of the first-hour protocol. A total of 17.5% (*n *= 14/80) thought there has been no effect, and none considered the protocol harmful for education. For ICU doctors the protocol did not bring either clear educational advantages or disadvantages. The variable life-adjusted display curves (The Finnish Intensive Care Consortium) have shown improvement in our patient care after the implementation of the first-hour protocol. However, we cannot determine whether it is a significant factor in our intensive care results.

## Conclusion

The first-hour protocol has helped us in resource management, start of the patients' intensive care and education of nursing staff.
